# An Updated Insight Into Molecular Mechanism of Hydrogen Sulfide in Cardiomyopathy and Myocardial Ischemia/Reperfusion Injury Under Diabetes

**DOI:** 10.3389/fphar.2021.651884

**Published:** 2021-10-26

**Authors:** Hai-Jian Sun, Zhi-Yuan Wu, Xiao-Wei Nie, Xin-Yu Wang, Jin-Song Bian

**Affiliations:** ^1^ Department of Pharmacology, Yong Loo Lin School of Medicine, National University of Singapore, Singapore, Singapore; ^2^ Department of Endocrinology, The First Affiliated Hospital of Shenzhen University (Shenzhen Second People’s Hospital), Shenzhen, China; ^3^ Department of Pharmacology, School of Medicine, Southern University of Science and Technology, Shenzhen, China; ^4^ National University of Singapore (Suzhou) Research Institute, Suzhou, China

**Keywords:** diabetes, diabetic cardiomyopathy, nitric oxide, hydrogen sulfide, ischaemia-reperfusion injury

## Abstract

Cardiovascular diseases are the most common complications of diabetes, and diabetic cardiomyopathy is a major cause of people death in diabetes. Molecular, transcriptional, animal, and clinical studies have discovered numerous therapeutic targets or drugs for diabetic cardiomyopathy. Within this, hydrogen sulfide (H_2_S), an endogenous gasotransmitter alongside with nitric oxide (NO) and carbon monoxide (CO), is found to play a critical role in diabetic cardiomyopathy. Recently, the protective roles of H_2_S in diabetic cardiomyopathy have attracted enormous attention. In addition, H_2_S donors confer favorable effects in myocardial infarction, ischaemia-reperfusion injury, and heart failure under diabetic conditions. Further studies have disclosed that multiplex molecular mechanisms are responsible for the protective effects of H_2_S against diabetes-elicited cardiac injury, such as anti-oxidative, anti-apoptotic, anti-inflammatory, and anti-necrotic properties. In this review, we will summarize the current findings on H_2_S biology and pharmacology, especially focusing on the novel mechanisms of H_2_S-based protection against diabetic cardiomyopathy. Also, the potential roles of H_2_S in diabetes-aggravated ischaemia-reperfusion injury are discussed.

## Introduction

The International Diabetes Federation has estimated that global diabetic prevalence might rise to 10.2% by 2030 and 10.9% by 2045, respectively (Saeedi et al., 2019). On a global scale, increasing epidemic of diabetes exerts serious economic and social implications in many countries, and 77% of diabetic individuals live in low and middle income countries (Standl et al., 2019). Apart from overt diabetic patients, 352.1 million people are estimated to be at risk of diabetes globally (also known as pre-diabetes) (Standl et al., 2019). Diabetic pandemic seems to be one of global public health problems that need to be solved urgently, especially considering the increasing number of deaths caused by diabetic complications (Ayensa-Vazquez et al., 2020; Magliano et al., 2020). Cardiovascular disorders are the most complications of diabetes with a serious impact on healthcare resources, and they are also the main causes of death in diabetic patients (International Hypoglycaemia Study Group, 2019). Diabetes-induced cardiovascular diseases encompass peripheral artery disease, cerebrovascular disease, coronary artery disease, heart failure, and cardiomyopathy (International Hypoglycaemia Study Group, 2019). A host of clinical studies have demonstrated that cardiovascular disease risks are 3-to-4-fold higher in diabetic patients (Xie et al., 2017). Diabetic cardiomyopathy, a unique type of cardiac damage, exhibits abnormal myocardial structures independent of hypertension, coronary artery disease, valvular heart disease or other known precipitating factors for heart failure (Rubler et al., 1972; Adeghate and Singh, 2014; Zamora and Villena, 2019). A population-based study has reported that diabetic patients are at a higher risk to develop diastolic or systolic dysfunctions when compared to normal individuals (Devereux et al., 2000; Bertoni et al., 2006). Diabetic cardiomyopathy has gained increased attention due to its high morbidity and poor prognosis (Oktay et al., 2000; Pikkemaat et al., 2017). Studies have confirmed that the pathogenesis of diabetic cardiomyopathy is multifactorial and mainly include, but not limited to, oxidative/nitrosative stress, impaired calcium homeostasis, mitochondrial dysfunction, and accumulation of advanced glycation end products (AGEs), as well as increased inflammation (Borghetti et al., 2018; Al Hroob et al., 2019). Although several anti-hyperglycemic drugs are commonly used for the treatment of diabetes, they can hardly improve diabetic cardiomyopathy (Borghetti et al., 2018). As a result, there is an urgent unmet medical need to discovery novel and effective therapies directly against diabetic cardiomyopathy.

Historically, hydrogen sulfide (H_2_S) was viewed as a toxic gas with foul smelling, but more and more studies have identified H_2_S as a member of the gasotransmitter family, along with nitric oxide (NO) and carbon monoxide (CO) (Beck and Pfeilschifter, 2020). In the cardiovascular system, H_2_S is endogenously generated by three endogenous enzymes, including cystathionine *β*-synthase (CBS), cystathionine *γ*-lyase (CSE), and 3-mercaptopyruvate sulfur transferase (3-MST)/cysteine aminotransferase (CAT) (Chen et al., 2020). Besides, H_2_S is also generated from d-cysteine by d-amino acid oxidase (DAO) (Wen et al., 2018). In the last several decades, the biological and pharmacological effects of H_2_S on various systems are gradually revealed after the measurement of brain H_2_S content in 1989 (Goodwin et al., 1989; Wang, 2002; Wang, 2012). Subsequently, H_2_S is emerging as an essential regulator in a diversity of physiological functions in the biological systems (Xie et al., 2016). Similar to NO and CO, H_2_S is critically involved in cardiovascular regulation (Testai et al., 2020). As the third gaseous signaling molecule, H_2_S is brought into the spotlight of cardiovascular field as H_2_S exhibits multiple cardiovascular protective effects, including vascular relaxation, pro-angiogenesis, anti-hypertension, anti-atherosclerosis, attenuation of oxidative stress and inflammation, as well as amelioration of myocardial ischemia-reperfusion injury (Gorini et al., 2020; Teoh et al., 2020). Coincidentally, accumulating evidence has suggested that H_2_S could modulate numerous molecular cascade events to ameliorate diabetes-induced cardiac damage (Kar et al., 2019a). Thereafter, we will highlight recent advances in the current understanding of the ability of H_2_S to relieve diabetic cardiomyopathy and diabetes-deteriorated ischaemia-reperfusion injury. Interactions of H_2_S with NO in diabetic cardiomyopathy are also described. Eventually, the possible challenges and directions of H_2_S-based pharmacological therapy in diabetic cardiomyopathy are overviewed and proposed.

## Pathophysiology of Diabetic Cardiomyopathy

In 1954, Lundbaek reported a distinct clinical entity with cardiac dysfunction in type 2 diabetic individuals (termed as diabetic cardiomyopathy), independent of hypertension and coronary artery disease (Lundbaek, 1954). Rubler et al. (1972) had observed glomerulosclerosis and heart failure in diabetic individuals, although these diabetic patients had no hypertension, coronary artery disease, valvular or congenital heart disease. A large population study found that the risk of heart failure hospitalization in diabetic patients was still higher even though all cardiovascular risk factors were well controlled (Rawshani et al., 2018). With rapid increases in diabetic incidence and prevalence, diabetic cardiomyopathy, an important cardiovascular complication of diabetes, is becoming one of the main causes of disability and death in diabetic individuals (Zhang et al., 2021). Of all diabetic complications, diabetic cardiomyopathy accounts for nearly 80% of death in diabetic individuals (Wang and Hill, 2015).

**FIGURE 1:**
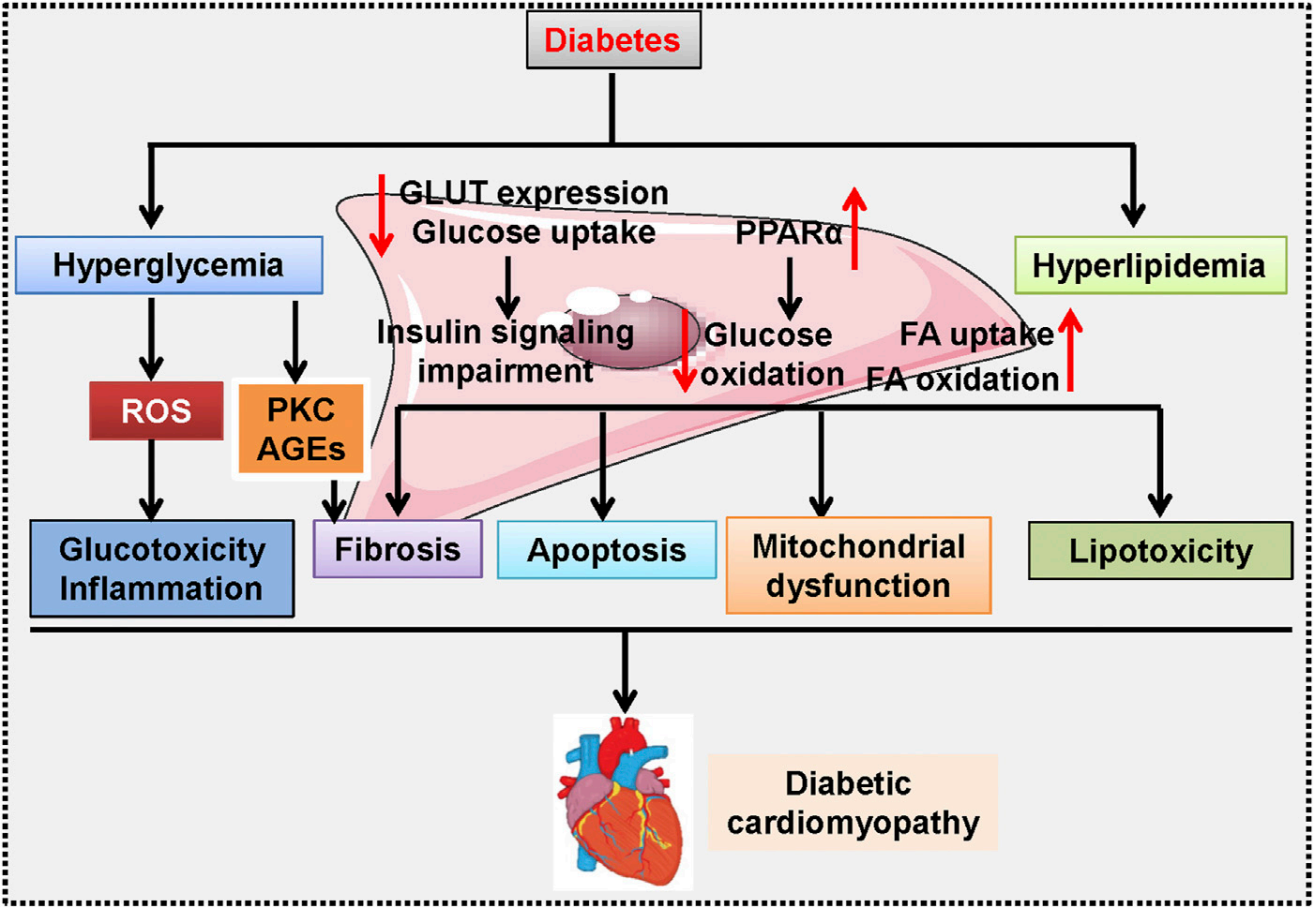
A proposed schematic diagram showing the signaling pathways involved in diabetic cardiomyopathy-related cardiac dysfunction. Hyperglycemia induces ROS overproduction and causes glucotoxicity and inflammation in the heart, and activation of PKC promotes AGEs formation, a critical event involved in diabetes-induced cardiac fibrosis. In cardiomyocytes, high glucose inhibits GLUT expression and glucose uptake, but upregulates PPARα to reduce glucose oxidation, thus resulting in insulin signaling impairment. Moreover, hyperlipidemia stimulates FA uptake and oxidation, and renders cardiomyocytes more sensitive to lipotoxicity. All these above changes synergistically contribute to fibrosis, apoptosis, mitochondrial dysfunction, and hypertrophy during the development and progression of diabetic cardiomyopathy. AGE: advanced glycation end products; FA: fatty acids; GLUT: Glucose transporters; PKC: Protein kinase C; PPARα: Peroxisome proliferator activated receptor α; ROS: reactive oxygen species.

Pathologically, diabetic cardiomyopathy is reflected by cardiac hypertrophy, myocardial apoptosis and fibrosis, and cardiac dysfunction (Ernande et al., 2011; Zhang and Sun, 2020). Numerous molecular mechanisms have been suggested to be involved in the development and progression of diabetic cardiomyopathy, such as oxidative stress, nitrosative/nitrative stress, inflammatory response, mitochondrial dysfunction, endoplasmic reticulum stress, impaired autophagy, cardiomyocyte apoptosis and death, and diabetic microangiopathy (Bugger and Abel, 2014; Varga et al., 2015; Kaludercic and Di Lisa, 2020). Additionally, experimental studies have shown that cardiac glycolipid toxicity, impaired insulin signaling, cardiac energetic impairment, activation of the renin–angiotensin system (RAS) and increased formation of angiotensin II (Ang II), cardiac autonomic neuropathy, reduced NO bioavailability, elevations in AGEs, and dysregulated cardiomyocyte calcium handling, play central roles in diabetic cardiomyopathy pathologies (Figure 1) (Pechánová et al., 2015; Jia et al., 2018a; Jia et al., 2018b; Dhalla and Shah, 2020; Komici et al., 2019; Zamora and Villena, 2019). Although our current understanding of the pathogenesis of diabetic cardiomyopathy is increasing, the etiology of diabetic cardiomyopathy is multifactorial and extremely complicated. There are still gaps in our current knowledge of the precise mechanisms involved in diabetic cardiomyopathy. As a result, more studies are required to deepen the comprehensive mechanisms that underlie the initiation and progression of diabetes-induced cardiac dysfunction, thereby leading to the development of newly effective targets or approaches to reduce the risk of diabetic patients developing life-changing complications, such as heart failure.

## Chemistry and Biochemistry of H_2_S

Previously, H_2_S is taken as a poisonous and occasionally lethal gas, and it might represent an industrial safety hazard since this colorless gas could be formed from the decomposition of various organic materials (Perna et al., 2011). Because of a special characteristic of rotten smell, H_2_S is believed to be a toxic byproduct of microbial metabolism in the atmosphere (Pan et al., 2017). H_2_S could be detected by the human nose at a level of 0.1 ppm, a dose of 400-fold lower than its toxic concentrations (Wang, 2002), while long-term exposure can render desensitization of the olfactory nerve to H_2_S (Li et al., 2009). H_2_S is readily dissolved in water due to its weak acid properties (p*K*a at 37°C, 6.76), and it will split to generate two dissociation states; the hydrosulphide anion (HS^−^, p*K*a 7.04), and sulphide anion (S^2−^, p*K*a 11.96) (Li et al., 2009). As a consequence, approximately 18.5% of the total sulphide exists as the undissociated acid and 81.5% as the HS^−^ and S^2−^ at physiological pH of 7.4 in aqueous solution (Dombkowski et al., 2004), which are in dynamic equilibrium in the following sequential reactions: H_2_S⇌HS^−^ +H_+_⇌S^2−^ + 2H^+^. Actually, the three equilibria in this formula represent the real dynamics of H_2_S in solution. It can be easily predicted that this equilibria will continuously shift to the left in an open system according to Le Chatelier’s Principle, thereby causing H_2_S to escape from the solution (Li and Lancaster, 2013). It has been shown that the half of H_2_S could be lost from solutions within 5 minutes in cell culture wells, 3 minutes in a bubbled tissue bath and an even shorter time in the Langendorff heart apparatus (DeLeon et al., 2012). Under aerobic conditions, the half-life time of H_2_S is 2.0, 2.8, and 10.0 min in murine hepatic, renal, and brain homogenates, respectively (Table 1) (Vitvitsky et al., 2012). One should also be aware that the actual concentration of H_2_S might vary in experimental systems containing headspace, and this may also depict to some extent the remarkable variations in H_2_S concentrations in cells, tissues or plasma (Furne et al., 2008; Whitfield et al., 2008; Shen et al., 2011; Kolluru et al., 2013). Whist it remains elusive whether the biological functions of H_2_S are mediated by H_2_S itself or its derived species, including S^2−^ or HS^−^, it is being accepted that HS− is a potential nucleophile that could react with different electrophilic cellular targets, such as nitrogen species (RSONS) (Hartle and Pluth, 2016). H_2_S is a well-known reducing agent that exhibits various chemical properties, such as its redox activity, acidity, and high nucleophilicity, allowing for reactions with multiple cellular targets (Hartle and Pluth, 2016). Although it is quite conceivable that the biochemical effects of H_2_S may be dependent on its chemical capacities (especially the reductive and nucleophilic properties), disentangling this chemistry seems to be more complicated (Li and Lancaster, 2013; Nagy et al., 2014).

**TABLE 1:** Half-life time of H2S.

Sources	Half-life time	References
Murine hepatic homogenates	2.0 min (Cysteine)	Vitvitsky et al. (2012)
Murine renal homogenates	2.8 min (Cysteine)	Vitvitsky et al. (2012)
Murine brain homogenates	10.0 min (Cysteine)	Vitvitsky et al. (2012)
Cell culture wells	5.0 min (Na_2_S·9H_2_O)	DeLeon et al. (2012)
A bubbled tissue bath	3.0 min (Na_2_S·9H_2_O)	DeLeon et al. (2012)
Langendorff heart apparatus	0.5 min (Na_2_S·9H_2_O)	DeLeon et al. (2012)

H_2_S is biologically active since its highly lipophilic characteristics allow it freely to penetrate into all types of the cell membranes without facilitation of membrane channels (Mathai et al., 2009). A famous pioneer in H_2_S research, Hideo Kimura, had demonstrated that H_2_S facilitated long-term potentiation in hippocampal tissues at the physiological concentration, pointing this gasotransmitter as an important neuromodulator (Abe and Kimura, 1996). Later, H_2_S is found to regulate both physiological and pathophysiological processes through a wide spectrum of signaling molecules (Kimura, 2020), such as reacting with superoxide anions (Chang et al., 2008), hypochlorite (Whiteman et al., 2005), hydrogen peroxide (Geng et al., 2004), peroxynitrite (Whiteman et al., 2004), metals (Li and Lancaster, 2013), thiol derivatives (Li and Lancaster, 2013), and NO (Ali et al., 2006; Whiteman et al., 2006) (Table 2). It is highly probable that the list of biomolecules affected by H_2_S will grow rapidly in the near future.

**TABLE 2:** Selected targets of H2S.

Selected targets	References
Superoxide anions	Chang et al. (2008)
Hypochlorite	Whiteman et al. (2005)
Hydrogen peroxide	Geng et al. (2004)
Peroxynitrite	Whiteman et al. (2004)
Metals	Li and Lancaster (2013)
thiol derivatives	Li and Lancaster (2013)
NO	Ali et al. (2006), Whiteman et al. (2006)

Since the discovery of H_2_S production in mammalian cells, many scientists have been working on the biological functions of H_2_S in this emerging field. The critical roles of H_2_S at its physiologically relevant concentrations on cardiovascular homeostasis have been well documented (Pan et al., 2017). Endogenous H_2_S levels are regulated preciously by its generation and elimination in health (Liu et al., 2012). It is important to determine the concentrations of H_2_S in cells, tissues, and blood samples before its prosperity in the regulation of physiological/pathophysiological functions. To date, several analytical methods are achieved to quantify H_2_S concentrations in organ tissues and blood, such as fluorescent probes (Zhang et al., 2020b), colorimetry (Sugahara et al., 2016), spectrophotometric analysis (Antoniou et al., 2018), headspace gas determination (Fonseca et al., 2013), polarographic sensor (Doeller et al., 2005), and liquid chromatography-mass spectrometry (Tan et al., 2017). However, different analysis methods have obtained inconsistent range of H_2_S concentrations (Ibrahim et al., 2021). According to the current literatures, the physiological concentrations of H_2_S in human/animal blood or tissues range from 15 nM to 300 μM *in vivo* (Huang et al., 2015; Hackfort and Mishra, 2016; Pan et al., 2017). Likewise, the physiological concentrations of H_2_S in plasma, serum, and cardiac tissues are considerably inconsistent (Table 3). The low concentration, short half-life time with fast catabolism, and high reactivity of H_2_S might be challenging for the determination of endogenous H_2_S *in vivo* (Ibrahim et al., 2021). Additionally, the wide range of H_2_S concentrations might result from variable detection methods as the different analysis technologies have distinct disadvantages, such as low sensitivity and specificity, complex preparation processes, and time-consuming procedures (Liu et al., 2012; Wu and Hu, 2018). The striking inconsistency in H_2_S levels under physiological conditions may lead to uncertainty for the exact mechanistic roles of H_2_S in physiological processes. Thence, it is essential to improve detection specificity and lower threshold limitations of these techniques, which may be helpful to obtain the actual physiological compartmentalization of H_2_S in cells, blood, and tissue.

**TABLE 3:** H_2_S concentrations in blood or cardiac tissues under physiological conditions.

Sample	Subject	H_2_S content	Reference
Serum	Mouse	~60 µM	Peleli et al. (2020)
	Mouse	9.46 ± 0.90 µM	Lv et al. (2020)
	Rat	24.75 ± 6.73 µM	Shi et al. (2019)
	Rat	~0.25 µM	Ma et al. (2019)
	Human	~200 µM	Renieris et al. (2020)
	Human	72.18 ± 10.66 µM	Burguera et al. (2020)
Plasma	Mouse	22.5 ± 1.9 µM	Bhatia et al. (2005)
	Mouse	0.2 ± 0.01 μM	Kasamatsu et al. (2020)
	Mouse	~17 µM	Behera et al. (2018)
	Mouse	~2 µM	Chen et al. (2018)
	Mouse	~1.5 µM	He et al. (2014)
	Rat	34.1 ± 0.7 µM	Li et al. (2005)
	Rat	~32 µM	Li and Xiao (2020)
	Rat	28.9 ± 1.4 µM	Mok et al. (2004)
	Rat	~40 µM	Yusuf et al. (2005)
	Rat	~12 µM	Possomato-Vieira and Gonçalves-Rizzi (2018)
	Human	54.1 ± 21.4 pg/ml	Jin et al. (2020)
	Human	67.15 ± 2.99 µM	Possomato-Vieira and Gonçalves-Rizzi (2018)
	Human	43.8 ± 20.82 µM	Sun et al. (2016)
	Human	~25 µM	Alyan et al. (2021)
	Human	70–125 μM	Karunya et al. (2019)
Heart	Mouse	~70 µM/mg protein	Peleli et al. (2020)
	Mouse	~7 µM/mg protein	Han et al. (2020)
	Mouse	~100 nM/mg protein	Shimizu et al. (2018)
	Rat	~60 µM/g protein	Sun et al. (2019c)
	Rat	~0.3 mM/mg protein	Ma et al. (2018)
	Rat	~0.2 µM	Guo et al. (2017b)

## Enzymatic Production and Metabolism of H_2_S

**FIGURE 2:**
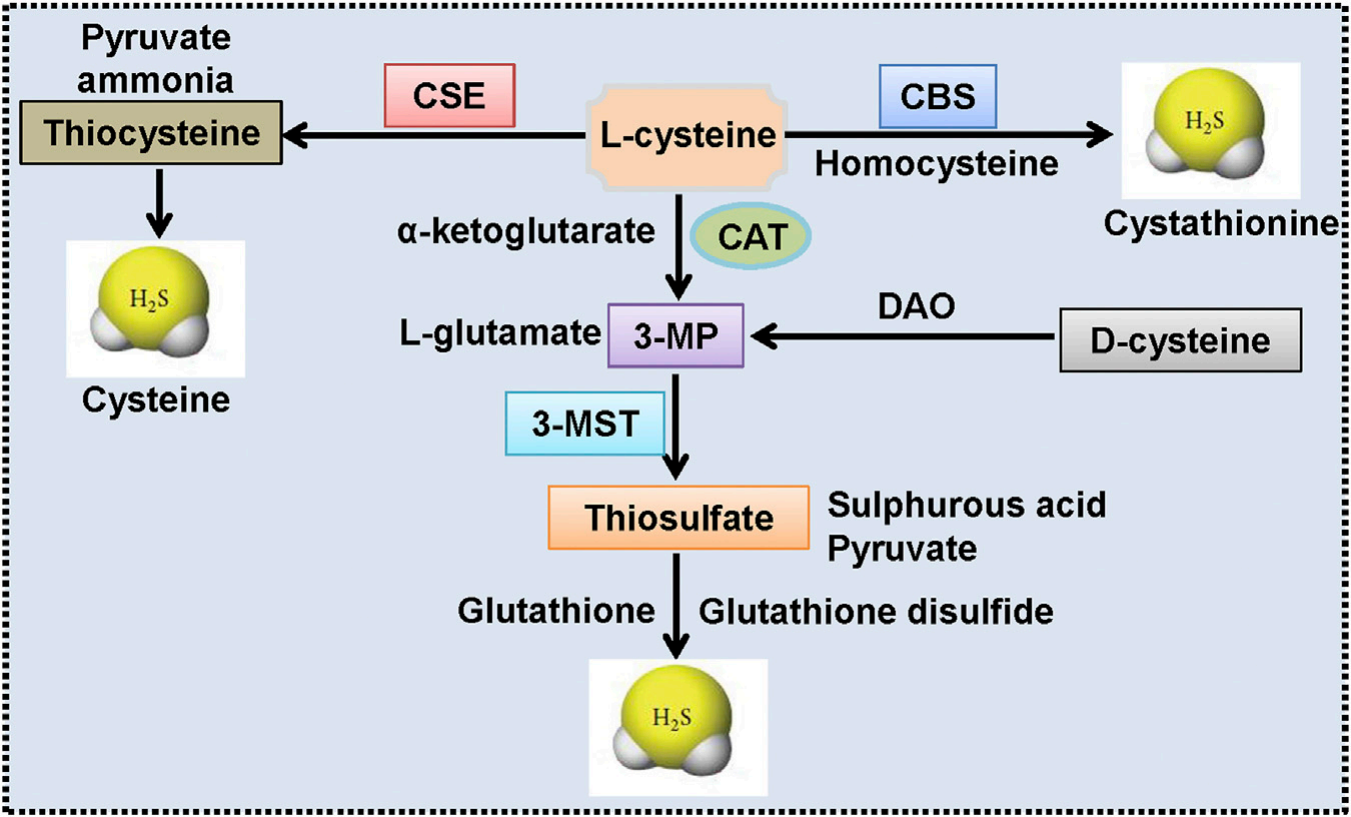
Endogenous synthesis pathways of H2S. CSE produces H_2_S by using l-cysteine as the main substrate, accompanied by the generation of ammonia, pyruvate, and thiocysteine. The latter would be catalyzed into H_2_S and cysteine. CBS-mediated H_2_S production is dependent on the transformation of homocysteine and l-cysteine to H_2_S and cystathionine. CAT catalyzes the conversion of l-cysteine and *α*-ketoglutarate to 3-MP and l-glutamate, and 3-MP is then catalyzed to sulfurous acid, pyruvate and thiosulfate by 3-MST. Thiosulfate is then reduced to H_2_S and glutathione disulfide by using reduced glutathione. Moreover, d-cysteine could be converted into 3-MP by DAO, and this reaction is also responsible for the formation of H_2_S in the mitochondria. 3-MP: 3-mercaptopyruvate; 3-MST, 3-mercaptopyruvate sulfurtransferase; CAT, cysteineamino transferase; CBS, cystathionine *β*-synthase; CSE, cystathionine *γ*-lyase; DAO, d-amino acid oxidase; H_2_S: hydrogen sulfide.

Endogenous H_2_S in mammalian systems is primarily produced by enzymatic or non-enzymatic pathways (Yu et al., 2014). The enzymatic pathways that generate H_2_S are mediated by CBS, CSE, and the coupling of 3-MST with CAT. The enzymatic pathways of H_2_S formation are illustrated in Figure 2. The intracellular distributions of H_2_S-generating enzymes are different in which CBS and CSE are strictly localized in the cytoplasm, whereas 3-MST and CAT are expressed in both the mitochondrial and the cytosol (but much higher in mitochondria) (Pan et al., 2017). Regarding tissue specificity localization, CSE is abundantly expressed in the cardiovascular system, whereas CBS expression predominates in the brain, liver, and kidneys under normal physiological conditions (Pan et al., 2017; Sun et al., 2019b). 3-MST and CAT are responsible for H_2_S formation in the brain and vascular endothelium (Shibuya et al., 2009; Chen et al., 2015). In spite of these observations, the relative contribution of each of these three enzymes to the tissue or circulating H_2_S levels remains elusive (Donnarumma et al., 2017). In addition to enzymatic pathways, H_2_S is also produced from inorganic polysulfides, organic polysulfides, and elemental sulfur, which are enriched in garlic (Benavides et al., 2007). Therefore, the healthy benefits of garlic may be associated with H_2_S production (Rose et al., 2018; Rodrigues and Percival, 2019). Actually, garlic extracts are effectively slowing the development and progression of atherosclerosis through the generation of H_2_S from S-allylcysteine and S-allylmercaptocysteine (Benavides et al., 2007; Budoff et al., 2009).

**FIGURE 3:**
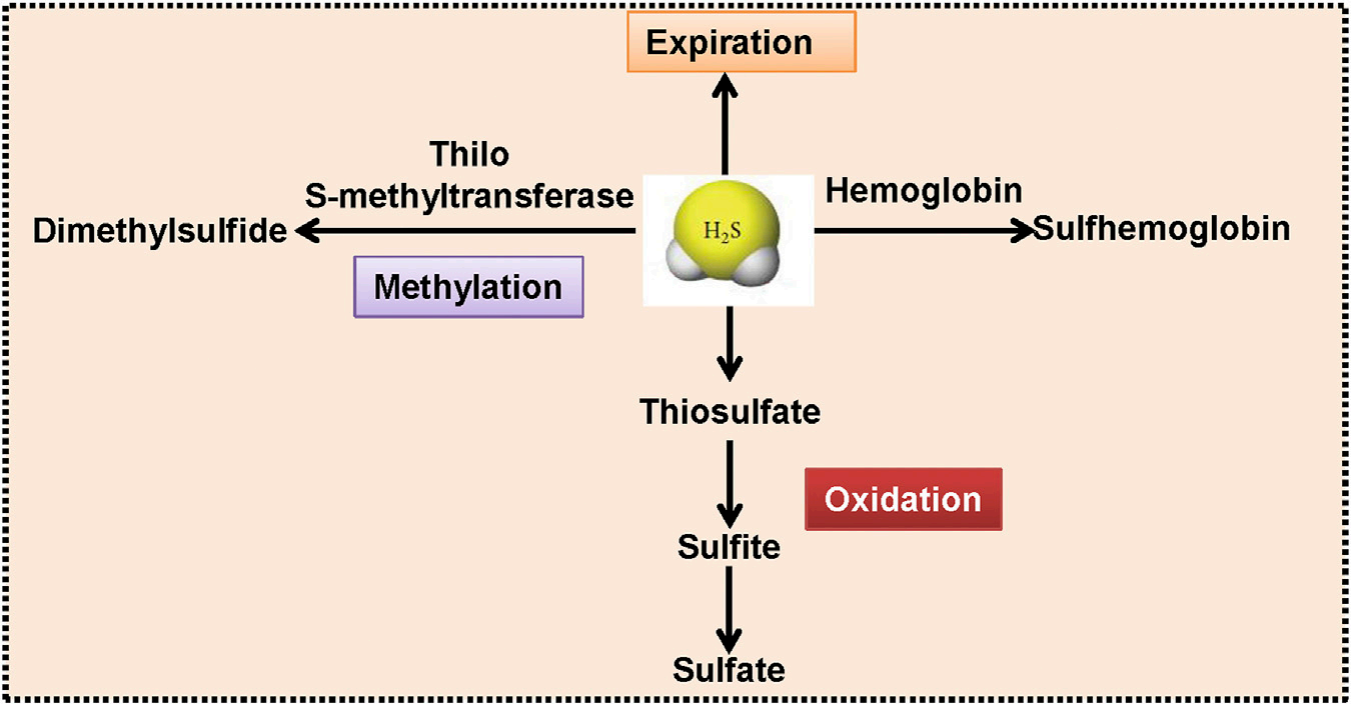
The proposed rotes for H2S degradation. With the aid of thiol S-methyltransferase, H_2_S is methylated into dimethylsulfide. H_2_S could be oxidized to thiosulfate, coupled with the production of sulfite and sulfate. Hemoglobin interacts with H_2_S, resulting in the generation of sulfhemoglobin. H_2_S can be released from the mammalian lungs. H_2_S, hydrogen sulfide.

The balance of synthesis and metabolism is essential for maintaining an appropriate concentration of H_2_S under physiological conditions. Endogenous H_2_S could be inactivated by several routes (Figure 3). First of all, H_2_S can be converted into thiosulfate through mitochondrial oxidative modification, thiosulfate is further transformed into sulfite and sulfate (Kimura, 2012). Cytosolic methylation is the second pathway to convert H_2_S to dimethylsulfide through thiol S-methyltransferase (Wang et al., 2020). Also, binding of hemoglobin to H_2_S contributes to the generation of sulfhemoglobin (Olson et al., 2014). The excessive H_2_S could be scavenged by metallo- or disulfide-containing molecules or glutathione disulfide, and could also be released from the lungs (Mani et al., 2014; Donnarumma et al., 2017; Rose et al., 2017; Cao et al., 2019). Although these biosynthesis and degradation pathways of H_2_S have only recently been identified, these findings will undoubtedly promote the clinical translational research of this gaseous transmitter in future studies. Recently, the physiological significance of H_2_S metabolites, such as persulfides, polysulfides, and other reactive sulfur species is emerging (Koning et al., 2015). Moreover, H_2_S and reactive sulfur species could coexist in biological system as they are normally interchangeable (Bolton et al., 2019). It is believed that reactive sulfur species might be responsible for at least some biological activities of H_2_S (Shimamoto and Hanaoka, 2015). However, the comparisons of the protective effects of H_2_S and its metabolites on cells or tissues remain unclear, which merits further research.

The potential of H_2_S metabolites as biomarkers is being appreciated since the levels of H_2_S metabolites in serum and urine may reflect renal disease severity, such as chronic kidney disease (Nakanishi et al., 2002; Koning et al., 2015). In chronic heart failure patients, plasma sulfate concentrations tend to be higher, and sulfate clearance is associated with favorable disease outcome (Koning et al., 2017). Although these observations are promising, it is clear that the recognition of H_2_S metabolites as disease biomarkers is not flawless. Meanwhile, the quantification of H_2_S metabolites in the ischemia-reperfusion conditions or in other cardiovascular debacles has yet to be fully elucidated. Therefore, large-population studies investigating the potential value of H_2_S metabolites as disease biomarkers are underway. Overall, our current understanding of H_2_S metabolites and how their dysfunction contributes to cardiovascular pathogenesis remains largely unclear, thereby requiring more studies.

## Pharmacology and Toxicology of H_2_S

It has been well documented that H_2_S is a crucial signaling molecule in the cardiovascular system because of its physiological and pathological significances in cardiovascular homeostasis (Liu et al., 2011; Pan et al., 2011; Sun et al., 2011; Liu et al., 2012; Pan et al., 2012; Wang et al., 2015b; Nagpure and Bian, 2016). Plasma and tissue levels of H_2_S are sophisticatedly controlled by its formation and catabolism in health (Elsey et al., 2010; Huang et al., 2015). Under pathological conditions, endogenous H_2_S levels and H_2_S-prodocuing enzyme activities/expressions are significantly altered (Pan et al., 2017; Sun et al., 2019a; Sun et al., 2019b). Both experimental and clinical evidence has revealed that perturbation of endogenous H_2_S system is obviously linked with the pathologies of cardiovascular disorders, including atherosclerosis, hypertension, diabetic cardiomyopathy, myocardial ischemia/reperfusion injury, endothelial dysfunction, and heart failure (Jiang et al., 2005; Wang et al., 2015c; Gao et al., 2015; Meng et al., 2015; Yang and Wang, 2015; Donnarumma et al., 2017; Kanagy et al., 2017; Li et al., 2017; Kar et al., 2019a). In renal physiology, H_2_S induces vasodilation, and increases renal blood flow and glomerular filtration rate, leading to an indirect increase of the urinary excretion of Na^+^ and K^+^ (Roy et al., 2012). H_2_S exhibits an inhibitory effect on specific Na^+^ and K^+^ transporters in the kidney, thus further increasing the excretion of such electrolyte into the urine (Xia et al., 2009). H_2_S acts as an oxygen sensor in the renal system, especially in medulla (Bełtowski, 2010). Also, H_2_S is found to inhibit renin release in rat models of renovascular hypertension (Lu et al., 2010). Hypertension-related nephropathy, a result of long-term hypertension, is the second leading cause of chronic kidney disease world (Hart and Bakris, 2010). The blood pressure lowering effects of exogenous H_2_S donors have been demonstrated in spontaneous hypertensive rats (Ahmad et al., 2014; Li et al., 2019a), angiotensin II-induced hypertension (Al-Magableh et al., 2015; Hsu and Tain, 2018; Chi et al., 2019), Nw-nitro-l-argininemethyl ester (l-NAME)-induced hypertension (Ji et al., 2014; Jin et al., 2017), and renovascular hypertension (Guo et al., 2017a; Feng et al., 2020). It is reasonable that renal protective effects of H_2_S are observed in these hypertensive models. Subsequent studies have suggested that suppression of ROS formation and epithelial sodium channel, upregulation of vascular endothelial growth factor might mediate renal protective effects of H2S in hypertension (Zhang et al., 2013; Ahmad et al., 2014; Holwerda et al., 2014; Wang et al., 2015a). Taken together, H_2_S serves as an ideal candidate for the prevention and treatment of hypertension-related renal damage.

The dose of H_2_S donor, sodium hydrosulfide (NaHS), ranging from 1.4 mg/kg/d to 56 mg/kg/d was used in animals, but 1 μM to 10 mM in cells (Wang et al., 2020). Similarly, the concentration of other H_2_S donors varies greatly in both animal and cell experiments (Wang et al., 2020). Here, it is thought-provoking to determine the exact pharmacological doses of H_2_S that are biologically effective or detrimental. Solving this problem may help to answer whether it is appropriate to use a dissolved exogenous H_2_S solution as a tool to determine the potential physiological function of endogenous H_2_S and its putative therapeutic applications. One should keep in mind that high micro-moles or even milli-moles solutions of dissolved H_2_S in organs or milieubathing cells have been repeatedly used in the literatures (Szabó, 2007; Nicholson and Calvert, 2010; Szabó et al., 2011). However, the solutions of dissolved H_2_S with high micro-moles or even milli-moles *in vitro* are above the concentrations of dissolved H_2_S found in blood and tissues during lethal H_2_S exposure *in vivo*. In addition, the level of toxicity varies greatly between different cell types, thus the physiological relevance of the data obtained after local or *in vitro* administration of H_2_S at concentrations of micro-moles or milli-moles is far from uncertain. Also, H_2_S is one of the most toxic mitochondrial poisons, even more toxic than cyanide on a mole-to-mole basis (Cooper and Brown, 2008). The activity of mitochondrial cytochrome c oxidase is diminished by a solution of dissolved H_2_S at a concentration ranging from 10 to 30 μM (Yong and Searcy, 2001; Leschelle et al., 2005). *In vivo* studies have shown that a severe depression in respiratory medullary neurons and/or cardiac contractility is observed in rodents and large mammals by infusing or inhaling H_2_S at levels yielding blood levels of gaseous H_2_S between 2 and 5 μM (Klingerman et al., 2013; Haouzi et al., 2014; Sonobe and Haouzi, 2016). A depression in cardiac contractility is produced by a solutions of H_2_S at concentrations above 50 μM on an isolated heart or cardiomyocytess (Geng et al., 2004; Sun et al., 2008; Yong et al., 2008). It is likely that this decrease in cardiac contractility is pathological and toxic at both the level of individual cardiac cells and the whole heart when exogenous H_2_S concentration reaches higher than the phyiological levels (Judenherc-Haouzi et al., 2016). In all, few micro-moles concentrations of dissolved H_2_S is toxic to most tissues *in vivo*, impacting vital respiratory and cardio-vascular functions (Haouzi, 2016). However, it seems that high levels of H_2_S are still administrated in the past, present and future research at the animal and cellular levels.

To date, it is merited to answer why the toxic levels of H_2_S are used in physiological studies. The rationality of using high levels (i.e., micro-moles or even milli-moles) of H_2_S to study and predict its physiological effects depends on the initial reports, where the concentration of H_2_S in blood or tissue is at high micromolar in the blood or tissues (Furne et al., 2008). Philippe Haouzi has reviewed that two main reasons could explain why high micro-moles of “endogenous” H_2_S are found in the blood and tissues (Haouzi, 2016). The first reason is related to the nature of the pools of sulfide present in the blood and in tissues, and the second reason for this error is linked with the methodology of measurement of the pool of diffusible/dissolved H_2_S in a biological milieu (Van de Louw and Haouzi, 2012). The subsequent studies have found that low levels of H_2_S donors *in vivo* are unlikely to increase the concentrations of H_2_S in tissues because of a fact that the majority of H_2_S in the blood after intravenous or intraperitoneal injections *in vivo* is immediately oxidized or combined with metallo-proteins (hemoglobin), dropping the level of free H_2_S to almost zero, unless the lethal levels are applied (Insko et al., 2009; Wintner et al., 2010; Haouzi and Klingerman, 2013; Klingerman et al., 2013). There is a very small margin between the concentration that does not produce an effect and the concentration that can kill. As reviewed by Szabo et al. (2011), the variable concentrations of H_2_S donors might result in the toxic levels or without chaging H_2_S concentrations in blood and tissues (Szabó, 2007). A growing number of studies support the “physiological” and protective roles of H_2_S by using solution of free H_2_S in the high micro-moles levels that are only found during a lethal in toxication *in vivo* (Haouzi and Klingerman, 2013). As such, high micro-moles or milli-moles levels of H_2_S donors are still used to elucidate the properties of endogenous H_2_S in those ongoing studies. In all, H_2_S donors regulate a multitude of biological processes at the typically used doses (so-called physiological effect), which should belong to the field of toxicology in many cases (Haouzi and Klingerman, 2013). Future studies are necessary to quantify the actual H_2_S concentration of each exogenous H_2_S donor used in the tissues and determine what effects are expected to be produced in keeping with sulfide toxicity and its rapid disappearance in the body or solutions.

### H_2_S: A Potential Cardioprotective Agent Against Diabetic Cardiomyopathy

Plasma H_2_S levels are remarkably decreased in diabetic patients and animals (King and Lefer, 2011; Guo et al., 2017b; Kesherwani et al., 2017; Suzuki et al., 2017). The levels of H_2_S in plasma, heart tissues, and urine tend to be lower in aging diabetic mice when compared to controls (Jin et al., 2015). Likewise, the myocardium expressions of H_2_S-generating enzymes, CSE and 3-MST, are significantly decreased in the heart samples of diabetic mice, whereas CBS levels are significantly elevated (Jin et al., 2015). Both circulating and myocardium levels of H_2_S are also reduced in high fat diet (HFD)-induced obese diabetic mice (Barr et al., 2015). In streptozotocin (STZ)-induced type 1 diabetic rats, a significant decrease in myocardial H_2_S concentration is also observed (Palamarchuk et al., 2020). These findings characterize a critical role for endogenous H_2_S dysfunction in the etiology of diabetic cardiomyopathy.

Recently, both *in vivo* and *in vitro* evidence has demonstrated the protective roles of H_2_S donors in diabetic cardiomyopathy (Table 4 and Table 5) (Wang, 2010; Patel et al., 2015). For instance, administration of exogenous H_2_S donors ameliorates cardiac dysfunction in diabetic rats and mice (Zhou et al., 2015; Sun et al., 2020d), which may be mediated by multiple signaling pathways or target proteins that are involved in myocardial hypertrophy (Cheng et al., 2016), cardiac fibrosis (Zhou et al., 2015), endoplasmic reticulum stress (Li et al., 2016), S-sulfhydration modification (Sun et al., 2020d), cardiomyocyte apoptosis, oxidative stress, and inflammation (Ye et al., 2018), NO production (Huang et al., 2016a), and autophagy (Wu et al., 2017).

**TABLE 4:** Molecular mechanisms of H_2_S donors in the treatment of diabetic cardiomyopathy at the animal level.

H_2_S donors	Animals	Main results	Ref.
NaHS (56 µM/kg/d; i.p.)	STZ-induced diabetic rats	Downregulation of the canonical Wnt and TGF-β1/Smad3 pathways and inhibition of myocardial hypertrophy/fibrosis	Yang et al. (2019)
NaHS (30 or 100 µM/kg; i.p.)	STZ-induced diabetic rats	Blockade of endoplasmic reticulum stress and inhibition of myocardial hypertrophy/fibrosis	Li et al. (2016)
NaHS (100 µM/kg; i.p.)	STZ-induced diabetic rats	Regulation of PKC/ERK1/2MAPK pathway, and inhibition of myocardial fibrosis/inflammation	Long et al. (2019)
NaHS (100 µM/kg; i.p.)	STZ-induced diabetic rats	Downregulation of the JAK/STAT signaling pathway, suppression of oxidative stress, inflammatory reaction, and cell apoptosis	Liu et al. (2018)
NaHS (100 µM/kg; i.p.)	STZ-induced diabetic rats	Attenuation of autophagy via the upregulation of the PI3K/AKT1 signaling pathway	Xiao et al. (2016)
NaHS (39 µM/kg; i.p.)	db/db mice	Inhibition of endoplasmic reticulum stress and suppression of myosin heavy chain 6 and myosin light chain 2 ubiquitination	Sun X. et al. (2020)
NaHS (28 µM/kg; i.p.)	STZ-induced diabetic rats	Upregulation of cardiac MLCK.	Yang et al. (2016a)
NaHS (100 µM/kg; i.p.)	STZ-induced diabetic rats	Inhibition of type I and III collagen, MMP-2 and MMP-9	Zheng et al. (2015)
NaHS (14 µM/kg; i.p.)	STZ-induced diabetic rats	Inhibition of inflammation, oxidative stress and apoptosis in the cardiac tissue via activating the Nrf2/ARE signaling pathway	Zhou et al. (2015)
SG-1002 (20 mg/kg; oral)	HFD-induced diabetic mice	Restoration of adiponectin levels and suppression of cardiac endoplasmic reticulum stress	Barr et al. (2015)
NaHS (100 µM/kg; i.p.)	STZ-induced diabetic rats	Attenuation of cardiac lipotoxicity and apoptosis via inhibiting endoplasmic reticulum stress	Guo et al. (2017b)
NaHS (100 µM/kg; i.p.)	STZ-induced diabetic rats	Inhibition of endoplasmic reticulum stress and mitochondrial apoptotic pathways	Yang et al. (2017a)
NaHS (80 µM/kg; i.p.)	db/db mice	Induction of VAMP3 degradation through Hrd1 S-sulfhydration and prevention of CD36 translocation	Yu et al. (2020)
NaHS (80 µM/kg; i.p.)	db/db mice	S-sulfhydration of USP8 and MuRF1 to regulate mitophagy	Sun Y. et al. (2020)
NaHS (14 µM/kg; i.p.)	STZ-induced diabetic mice	Induction of FoxO1 phosphorylation and nuclear exclusion	Ye et al. (2018)
NaHS (56 µM/kg; i.p.)	STZ-induced diabetic rats	Negative regulation of Thioredoxin-interacting protein-mediated NLRP3 inflammasome activation	Jia et al. (2020)
NaHS (56 µM/kg; i.p.)	STZ-induced diabetic rats	Suppression of iNOS activity and expression and inhibition of oxidative stress injury	Yang et al. (2017b)
NaHS (5 mg/kg; s.c.)	STZ-induced diabetic rats	Reversing disordered calcium-handling system in sarcoplasmic reticulum	Cheng et al. (2016)
NaHS (100 µM/kg; i.p.)	db/db mice	Facilitating autophagosome content degradation, and increasing Keap-1 expression by suppressing its ubiquitylation	Wu et al. (2017)

These documents were searched using Pubmed up to May 2021 with the search terms: (diabetic, diabetes, hyperglycemia, OR high glucose) AND (heart OR cardiomyopathy). After that, the titles and abstracts of all possible relevant papers were screened.

**TABLE 5:** Molecular mechanisms of H_2_S donors in the treatment of diabetic cardiomyopathy at the cellular level.

H_2_S donors	Cells	Main results	Ref.
NaHS (100 µM)	H9c2 cells	Facilitating ubiquitin aggregates clearance via autophagy	Wu et al. (2017)
NaHS (50, 100 or 200 µM)	Neonatal rat cardiomyocytes	Reversing disordered calcium-handling system in sarcoplasmic reticulum	Cheng et al. (2016)
GYY4137 (50, 100 or 200 µM)	H9c2 cells	Suppression of HG-induced cytotoxicity by activation of the AMPK/mTOR signal pathway	Wei et al. (2014)
NaHS (400 μM)	H9c2 cells	Inhibiting the leptin-p38 MAPK pathway	Zhuang et al. (2014)
NaHS (400 μM)	H9c2 cells	Inactivation of the NF-κB and IL-1β pathways	Xu et al. (2015)
NaHS (400 μM)	H9c2 cells	Inactivation of the p38 MAPK and ERK1/2 pathways and prevention of oxidative stress	Xu et al. (2013)
NaHS (400 μM)	H9c2 cells	Inhibition of the p38 MAPK pathway	Guo et al. (2013)
NaHS (400 μM)	H9c2 cells	Suppression of inflammation and cytotoxicity via inhibition of p38 MAPK/NF-κB signaling	Huang et al. (2016a)
NaHS (400 μM)	H9c2 cells	Suppression of inflammation and apoptosis by inhibiting the TLR4/NF-κB/NLRP3 pathway	Huang et al. (2016b)
NaHS (100 μM)	Neonatal rat cardiomyocytes and H9c2 cells	Suppression of cell apoptosis and oxidative stress by inhibiting the Wnt/beta-catenin pathway	Zhang and Ye, (2019)
GYY4137 (50, 100 or 200 µM)	H9c2 cells	Suppression of cell apoptosis and oxidative stress by inhibiting the STAT3/HIF-1α pathway	Li et al. (2019b)
GYY4137 (100 μM)	Neonatal rat cardiomyocytes	Induction of FoxO1 phosphorylation and nuclear exclusion	Ye et al. (2018)
NaHS (100 μM)	Neonatal rat cardiomyocytes	S-sulfhydration of USP8 and MuRF1 to regulate mitophagy	Sun Y. et al. (2020)
NaHS (100 μM)	Neonatal rat cardiomyocytes	Induction of VAMP3 degradation through Hrd1 S-sulfhydration and prevention of CD36 translocation	Yu et al. (2020)
GYY4137 (100 μM)	H9c2 cells	Regulation of endoplasmic reticulum-mitochondria cross-talk to inhibit cell apoptosis	Yang et al. (2017a)
NaHS (100 μM)	AC16 cardiomyocytes	Increasing cell viability and preventing lipid deposition through endoplasmic reticulum stress inhibition	Guo et al. (2017b)
NaHS (100 μM)	Neonatal rat cardiomyocytes	Inhibition of cell apoptosis by inhibiting JNK and p38 MAPK pathways and activating PI3K/Akt signaling	Zhou et al. (2015)
GYY4137 (100 nM)	Neonatal rat cardiac fibroblasts	Inhibition of ROS/ERK1/2 signaling pathway, and MMP-2 and 9 expressions	Zheng et al. (2015)
NaHS (100 μM)	Neonatal rat cardiomyocytes	H_2_S regulates MuRF1 S-sulfhydration at Cys44 to prevent myocardial degradation	Sun X. et al. (2020)

These documents were searched using Pubmed up to May 2021 with the search terms: (diabetic, diabetes, hyperglycemia, OR high glucose) AND (heart OR cardiomyopathy). After that, the titles and abstracts of all possible relevant papers were screened.

Induction of endogenous H_2_S production is also beneficial for diabetic cardiomyopathy. Diallyl trisulfide is reported to afford a protection against high glucose-provoked cardiomyocyte apoptosis by stimulating the formation of CSE-derived H_2_S (Tsai et al., 2015). Metformin, one of the most widely prescribed insulin sensitizer, might benefit diabetic cardiomyopathy by enhancing H_2_S production in the heart (Wiliński et al., 2013). Phosphodiesterase 5 (PDE5) inhibitors have powerful protective effects against diabetic cardiomyopathy, and this may be dependent on H_2_S generation (Das et al., 2015). Exercise training mitigates HFD-induced diabetic cardiomyopathy *via* promoting cardiac H_2_S biosynthesis and subsequent prevention of cardiomyocyte pyroptosis (Kar et al., 2019b). Pretreatment with curcumin alleviates pathological morphological damage in myocardium tissues from diabetic rats, and the beneficial effects of curcumin might be related with increased myocardium CSE and H_2_S levels (Tong et al., 2018). These exciting findings suggest the cardioprotective actions of H_2_S-based therapeutics in preclinical models of diabetic cardiomyopathy. Modulation of endogenous H_2_S production or application of exogenous H_2_S donors might serve as promising therapies for the management of diabetic cardiomyopathy. Later, we will provide a comprehensive overview on the critical molecular/cellular mechanisms that mediate H_2_S-induced favorable effects on diabetic cardiomyopathy. Emerging evidence suggests that H_2_S-mediated signaling transduction is correlated with production of NO (Paul and Snyder, 2012; Polhemus and Lefer, 2014; Li et al., 2018; Sun et al., 2020b). Thus, the current roles of interactive functions of H_2_S and NO in diabetic cardiomyopathies are also elaborated in this review.

## Inhibition of Myocardial Fibrosis and Hypertrophy

A previous study has found that administration of GYY4137 diminishes rat neonatal cardiac fibroblast migration exposed to high glucose (Zheng et al., 2015). The underlying mechanisms may involve inhibition of reactive oxygen species (ROS)/ERK1/2 signaling, indicating that H_2_S antagonizes cardiac fibrosis in diabetic cardiomyopathy by regulating redox homeostasis (Zheng et al., 2015). Consistent with this finding, NaHS prevents cardiac hypertrophy/fibrosis and ameliorates left ventricular dysfunction in STZ-induced diabetic rats by activating the nuclear factor erythroid 2-related factor 2 (Nrf2)/antioxidant response element (ARE) signaling pathway, accompanied by upregulations of antioxidant proteins haem oxygenase-1 (HO-1) and Nad(p)h: quinone oxidoreductase 1 (NQO1) in myocardium tissues from diabetic rats (Zhou et al., 2015). Exogenous H_2_S reverses myocardial hypertrophy and fibrosis through suppressing cardiac levels of myosin heavy chain 6 and myosin light chain 2 ubiquitination in db/db mice (Sun et al., 2020d). Compared with diabetic rats, the left ventricular hemodynamic parameters and myocardial ultrastructure changes (myocardial hypertrophy and fibrosis) are obviously normalized in diabetic animals treated with NaHS, which may be associated with upregulation of cardiac myosin light chain kinase (MLCK) and downregulation of serum creatine kinase MB isozyme (CK-MB) (Yang et al., 2016a).

The myocardial expressions of pro-fibrotic factors, including matrix metalloprotease 2 (MMP-2), tissue inhibitor of metalloproteinase 2 (TIMP-2), collagens, transforming growth factor (TGF)-β1/SMAD family member 3 (Smad3) signaling pathway, are strikingly changed in diabetic rats (Yang et al., 2019). However, an exogenous H_2_S donor, NaHS, ameliorates diabetes-induced myocardial fibrosis by inhibition of the Wnt and TGF-β1/Smad3 pathway and downregulation of myocardial collagen overproduction (Yang et al., 2019). Administration of NaHS for 8 weeks prevents myocardial hypertrophy and collagen deposition in STZ-induced diabetic rats by suppression of endoplasmic reticulum stress (Li et al., 2016). Inhibition of the protein kinase C (PKC)/extracellular regulated protein kinase 1/2 (ERK1/2) signaling pathway is required for H_2_S to ameliorate myocardial fibrosis in diabetic rats induced by STZ (Long et al., 2019). Liu et al. (2018) have found that the effects of NaHS on reversing diabetic myocardial fibrosis may be associated with blockade of the Janus kinase/signal transducer and activator of transcription (JAK/STAT) signaling pathway. Hematoxylin and eosin staining and Masson staining results have shown that NaHS is able to improve myocardial hypertrophy and fibrosis in diabetic rats induced by STZ, and the underlying mechanism may be dependent on activation of the phosphatidylinositol 3-kinase (PI3K)/Akt signaling pathway and following autophagy inhibition (Xiao et al., 2016). These above observations evidently suggest that H_2_S donors function as an attractive alternative for the prevention and treatment of diabetes-provoked myocardial hypertrophy and fibrosis (Kang et al., 2020).

## Suppression of Endoplasmic Reticulum Stress

**FIGURE 4:**
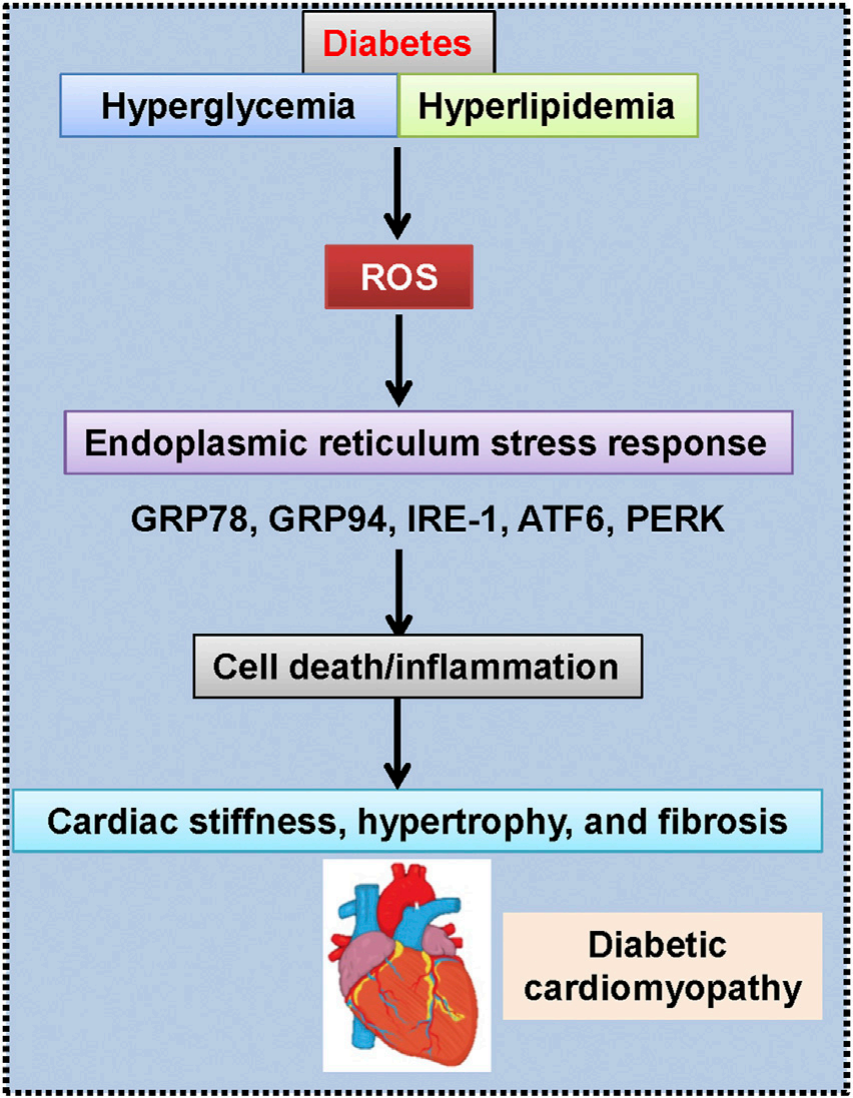
Engagement of endoplasmic reticulum stress in diabetic cardiomyopathy. ATF6, activating transcription factor 6; GRP78, glucose-regulated protein-78; GRP94, glucose-regulated protein-94; IRE-1, inositol-requiring kinase-1; PERK, protein kinase R-like ER kinase; ROS, reactive oxygen species.

Nowadays, the involvement of endoplasmic reticulum stress in the development and progression of diabetic cardiomyopathy is well accepted (Figure 4) (Xu et al., 2012). Given the important roles of endoplasmic reticulum stress in the pathologies of diabetic cardiomyopathy, inhibition of endoplasmic reticulum stress would be a therapeutic strategy for intervention of diabetic cardiomyopathy. Interestingly, multiple lines of evidence have proved that the protective effects of H_2_S against diabetic cardiomyopathy are related to inhibition of endoplasmic reticulum stress. For instance, pretreatment of AC16 cardiomyocytes with NaHS could retard palmitic acid-induced cardiac lipotoxicity and apoptosis, which is similar to the effects of an endoplasmic reticulum stress inhibitor 4-phenylbutyric acid (Guo et al., 2017b). The expressions of endoplasmic reticulum stress-associated proteins, including glucose-regulated protein (GRP78), C/EBP-homologous protein (CHOP), and caspase-12, are incremented in diabetic heart tissues, while H_2_S suppresses diabetes-induced endoplasmic reticulum stress and abrogates myocardial hypertrophy and myocardial collagen deposition in diabetic rats (Li et al., 2016). In line with this, intraperitoneal injection of NaHS improvesc cardiac dysfunction and myocardial ultrastructure damage by suppressing the mRNA expressions of endoplasmic reticulum stress markers, such as CHOP, GRP78 and caspase 12 (Yang et al., 2016b). Furthermore, the upregulated expressions of endoplasmic reticulum stress-related proteins and mitochondrial apoptotic proteins in diabetic cardiac tissues are reduced to normal levels in the presence of NaHS (Yang et al., 2017a).

Deletion of endogenous H_2_S is critically involved in diabetes-induced cardiac damage, whereas exogenous H_2_S donor NaHS improves diabetic cardiomyopathy though inhibition of endoplasmic reticulum stress (Guo et al., 2017b). Molecular experiments have revealed that H_2_S therapy (SG-1002) is capable of relieving HFD-induced cardiomyopathy *via* suppressing cardiac endoplasmic reticulum stress and restoring cardiac adiponectin levels (Barr et al., 2015). These above findings indicate that H_2_S donors are postulated to serve as novel cardioprotective agents for diabetic cardiomyopathy due to its strong suppression of endoplasmic reticulum stress-dependent apoptosis pathway in the heart.

## Protein S-Sulfhydration

S-sulfhydration (also called S-persulfidation or Persulfidation), a newly post-translational modification by generating a hydropersulfide moiety (–SSH) or polysulfide in specific cysteine residues of target proteins, is discovered to mediate most of the cellular functions induced by H_2_S (Paul and Snyder, 2012; Paul and Snyder, 2015; Zhang et al., 2017; Sun et al., 2021). Accordingly, several S-sulfhydrated proteins are involved in H_2_S-mediated cardiovascular benefits in diabetes. HMG-CoA reductase degradation protein (Hrd1), an endoplasmic reticulum transmembrane E3 ubiquitin ligase, is vitally involved in ubiquitylation of various substrate proteins and subsequent protein trafficking (Doroudgar et al., 2015). Vesicle-associated membrane protein 3 (VAMP3) is predominantly expressed in the heart and plays a key role in controlling intracellular membrane trafficking and exocytosis (Haglund and Dikic, 2012). Emerging evidence has shown that triacylglycerol accumulation within the myocardium may be responsible for the development of diabetes-induced heart failure, and this process is mainly modulated by transport protein fatty acid translocase CD36 (Chandler et al., 2003; Coort et al., 2004). VAMP3 contributes to CD36 exocytosis in cardiomyocytes, thus leading to increased myocardial long-chain fatty acid uptake and triacylglycerol accumulation (Pulido et al., 2011). On these grounds, Zhang and colleagues hypothesized that H_2_S induced VAMP3 degradation through Hrd1 S-sulfhydration and prevented CD36 translocation, thereby alleviating lipid toxicity in the heart tissues of db/db diabetic mice (Yu et al., 2020). As expected, the authors have demonstrated that exogenous H_2_S restores Hrd1 expression by inducing its S-sulfhydration at Cys115 (Yu et al., 2020). VAMP3 ubiquitylation and degradation induced by H_2_S is able to delay CD36 exocytosis in cardiomyocytes and reduce droplet formation in the heart tissues from db/db mice (Yu et al., 2020). This study leads to a novel finding that H_2_S upregulates Hrd1 expression through its S-sulfhydration at Cys115 and results in VAMP3 ubiquitylation and the stability of membrane CD36 expression in cardiomyocytes, which is necessary to prevent myocardial long-chain fatty acid uptake and lipid droplet formation in diabetic heart tissues (Yu et al., 2020). Afterwards, the same group has identified that H_2_S promotes the S-sulfhydration of ubiquitin specific peptidase 8 (USP8) and muscle RING finger-1 (MuRF1), thereby preventing cardiac structural injury in diabetes (Sun et al., 2020d; Sun et al., 2020e). These observations suggest that protein S-sulfhydration is believed to participate in the protective roles of H_2_S donors in diabetes-induced cardiomyopathy. In this regard, more S-sulfhydrated proteins will be identified to be responsible for the therapeutic effects of H_2_S in the context of diabetic cardiomyopathy.

## Inhibition of Cardiomyocyte Apoptosis, Oxidative Stress, and Inflammation

Evidence from cell cultures, animal models, and clinical studies has confirmed that H_2_S grants an anti-diabetic cardiomyopathy characteristic through anti-inflammation, anti-apoptosis, anti-oxidant stress pathways (Powell et al., 2018). Pretreatment of H9c2 cells with NaHS obviously counteracts high glucose-induced cardiac cell apoptosis and inflammation, as evidenced by measurement of caspase-3, inducible nitric oxide synthase (iNOS), cyclooxygenase 2 (COX-2), interleukin 1β (IL-1β), and IL-6 (Huang et al., 2016a). Inhibition of p38 MAPK/NF-κB signaling might mediate these cytoprotective effects of H_2_S in diabetes-induced cardiac apoptosis and inflammation (Huang et al., 2016a). Moreover, NaHS inactivated the toll-like receptor 4 (TLR4)/nuclear factor kappa-B (NF-κB) pathway to attenuate high glucose-induced nucleotide-binding oligomerization domain-like receptor protein 3 (NLRP3) inflammasome activation and cardiotoxicity in H9c2 cells (Huang et al., 2016b).

In addition, inactivation of Wnt/β-catenin signaling pathway and the signal transducer and activator of transcription 3 (STAT3)/hypoxia-inducible factor-1α (HIF-1α) pathway, upregulations of HO-1 and NQO1, induction of forkhead box O1 (FoxO1) phosphorylation and nuclear exclusion, are proposed to be main mechanisms for H_2_S donors to attenuate hyperglycemia-induced myocardial apoptosis and oxidative stress (Ye et al., 2018; Li et al., 2019b; Zhang and Ye, 2019). Moreover, H_2_S donor NaHS is reported to lessen hyperglycemia-induced inflammation, apoptosis, and oxidative stress in cardiac tissues by inhibiting c-Jun N-terminal kinase (JNK)/p38 mitogen-activated protein kinase (MAPK) pathways and activating PI3K/Akt signaling (Zhou et al., 2015). In the heart tissues, hyperglycemia significantly induces cardiac dysfunction with concomitant increases in redox perturbation and inflammatory reactions (Jia et al., 2020). However, treatment with NaHS significantly ameliorates these abnormalities, whereas treatment with propargylglycine (a specific inhibitor of H_2_S) further aggravated such alterations (Jia et al., 2020). These studies highlight the protective roles of H_2_S in diabetes-induced cardiac damage, and its potential mechanism may involve the negative regulation of cardiomyocyte apoptosis, oxidative stress, and inflammation.

Different from apoptosis, pyroptosis is a special pattern of cell death whereby the canonical pathway is reliant on caspase-1 and the noncanonical pathway is specifically dependent on caspase-11 (Bergsbaken et al., 2009). Initially found in monocytes and macrophages, pyroptosis is recently found to be involved in pathological inflammation-related cell death (Miao et al., 2010). NLRP3 inflammasome activates caspase-1 and results in cell swelling and lysis, and consecutive release of IL-1β and IL-18, characteristics of pyroptosis (Bergsbaken et al., 2009). Pyroptosis and its downstream inflammatory cytokines are closely linked with cardiovascular diseases, including diabetic cardiomyopathy (Kar et al., 2019a). NLRP3-dependent pyroptosis is detected in diabetic heart tissues, and it participates in the pathogenesis of diabetic cardiomyopathy (Luo et al., 2014; Luo et al., 2017). As a cardioprotective gaseous signaling molecule, H_2_S donor treatment suppresses caspase-1 activity and caspase-1 recruitment via the apoptosis-associated speck-like protein containing A CARD (ASC) adapter protein in a rat model of myocardial ischemic and inflammatory injury (Toldo et al., 2014). Recently, induction of H_2_S biosynthesis by exercise ameliorates HFD-induced cardiac structural and metabolic by inhibition of pyroptotic signaling (Kar et al., 2019b). However, further evidence is required to extrapolate the benefits of H_2_S donors-induced inhibition of pyroptosis on diabetic cardiomyopathy.

### Inhibition of the Leptin-p38 Mitogen Activated Protein Kinase Pathway

Leptin, a 16-kD hormone primarily secreted by adipocytes, exhibits a wide range of biological activities through binding to the leptin receptors (Friedman and Halaas, 1998). It is found that diabetes-induced cardiomyocyte hypertrophy may be mediated by leptin and its receptors (Majumdar et al., 2009). Aberrant expression of p38 MAPK is detected in the heart tissues from diabetic animals, and inhibition of p38 MAPK markedly delays the progression of diabetic cardiomyopathy (Zuo et al., 2019). In light of its capacity to facilitate diabetic cardiomyopathy development, p38 MAPK serves as an attractive target for the treatment of diabetic cardiomyopathy (Li et al., 2014). Indeed, inhibition of the p38 MAPK pathway is responsible for H_2_S-mediated protective effects against high glucose- or oxorubicin-induced cardiotoxicity in H9c2 cells (Guo et al., 2013; Xu et al., 2013).

Venkatesh and coworker have confirmed that leptin and its downstream p38 MAPK signaling pathway contribute to endothelin-1- and angiotensin-II-induced cardiomyocyte hypertrophy (Rajapurohitam et al., 2012). However, it remains to be investigated whether the leptin-p38 MAPK pathway contributed to high glucose-caused cardiomyocyte insult, and whether the pharmacological actions of H_2_S are related to this signaling pathway. To answer this, Liao’ group measured the leptin, leptin receptors, and p38 MAPK expression levels in high glucose-challenged cardiomyoblasts (Zhuang et al., 2014). Results have shown that the expression levels of leptin and its receptors are significantly enhanced in cardiomyoblasts (H9c2 cells) upon exposure to high glucose (35 mM) for 24 h, while these increases are markedly attenuated by pretreatment with NaHS (400 μM), a donor for H_2_S (Zhuang et al., 2014). Additionally, high glucose-induced increased phosphorylated p38 MAPK expression is reversed by either NaHS or leptin antagonist, suggesting the involvement of the leptin-p38 MAPK axis in high glucose-elicited cardiomyoblast injury (Zhuang et al., 2014). Importantly, H_2_S-mediated inactivation of the leptin-p38 MAPK pathway mitigates high glucose-evoked apoptosis, ROS generation, and mitochondrial membrane potential loss in H9c2 cells (Zhuang et al., 2014). Altogether, this study indicates that exogenous H₂S donors protect H9c2 cells against high glucose-induced injury at least in part by inactivating the leptin-p38 MAPK signaling pathway.

### Crosstalk With NO

A plethora of studies have revealed the physiological and/or pathological roles of H_2_S and NO in various systems during the last several decades (Lee and Yen, 2009; El-Mas et al., 2012; Nagpure and Bian, 2016; Magierowski et al., 2018; Wu and Hu, 2018; Mukherjee and Corpas, 2020; Xuan et al., 2020). Importantly, studies on the interaction of H_2_S and NO in diabetic cardiomyopathy are gradually emerging.

Several studies have disclosed that H_2_S regulates NO activities in the cardiovascular system (Zhang et al., 2020a), suggesting that H_2_S might exhibit cardioprotective effects via NO signaling. A phase I clinical trial has demonstrated that administration of a novel H_2_S prodrug, SG1002, increases H_2_S and NO levels in both healthy controls and heart failure patients (Polhemus et al., 2015). Exposure of endothelial cells with H_2_S stimulates NO production from endothelial nitric oxide synthase (eNOS) phosphorylation at Ser 1177 through an Akt-dependent mechanism (Predmore et al., 2011). CSE knockout mice are more susceptible to transverse aortic constriction as CSE-deficient mice display greater cardiac dilatation and dysfunction (Kondo et al., 2013). By contrast, cardiac-specific CSE overexpression or H_2_S therapy with SG-1002 restores cardiac structure and function after transverse aortic constriction through activation of the vascular endothelial growth factor-Akt-eNOS-NO-cGMP pathway (Kondo et al., 2013). Furthermore, CSE-deficient mice exhibit diminished eNOS activities, dysfunctional NO levels, and are more vulnerable to myocardial and hepatic ischemia reperfusion injury (King et al., 2014).

Acute H_2_S therapy improves myocardial and hepatic ischemia reperfusion injury by restoring eNOS function and NO bioavailability (King et al., 2014). NaHS treatment restores the impaired endothelial-dependent relaxation of the aorta in diabetic rats by a NOS-dependent mechanism as inhibition of NOS activity by l-NAME abolishes NaHS-mediated vasodilatation in diabetic aortas (Dorofeyeva et al., 2020a). These findings suggest that H_2_S-mediated cytoprotective signaling may be dependent in large part on eNOS-derived NO formation. As a pro-inflammatory factor and NO producer, iNOS express levels are upregulated in high glucose-incubated H9c2 cells, and H_2_S counteracts high glucose-induced inflammation and cytotoxicity via inhibition of iNOS expression (Huang et al., 2016a). In keeping with this, myocardial iNOS activities and expressions are significantly elevated in diabetic rats, which are suppressed by treatment with NaHS, a donor for H_2_S (Yang et al., 2017b). In sharp contrast, modulation of H_2_S synthesis stimulates eNOS activities and NO production in the heart tissues and aortic rings, thus recovering endothelium-dependent relaxation and arterial elastance, as well as cardiac dysfunction in diabetic rats (Dorofeyeva et al., 2021). Injection of STZ elicits reductions in myocardial CSE expression/activity, H_2_S and NO levels in rats, and moxonidine treatment abrogated cardiovascular dysfunction in diabetic rats through restoring cardiac H_2_S and NO levels (El-Sayed et al., 2016). These above findings imply that H_2_S confers a protective effect on the myocardium in diabetic animals, which may be associated with the regulations of iNOS, eNOS, and NO production.

It should be mentioned here that chemical interaction of H_2_S with NO might yield nitroxyl (HNO) (De Witt et al., 2001; Hrabie and Keefer, 2002; Cortese-Krott et al., 2014), one-electron-reduced product of NO, and HNO is emerging as a potential pharmacological agent for various diseases by targeting numerous signal pathways (Irvine et al., 2008; Switzer et al., 2009; Sun et al., 2020a; Sun et al., 2020b). An interesting study has shown that CSE-produced H_2_S scavenges vascular NO levels in peripheral arteries and contributes to blood pressure regulation (Szijártó et al., 2018). Pretreatment of vascular rings from CSE-deficient mice with H_2_S donor ameliorates endothelial vasorelaxant response by decreasing NO levels and restoring HNO levels in arteries (Szijártó et al., 2018). Our group recently found that mixture of H_2_S donor NaHS and NO donor sodium nitroprusside (SNP) gives rise to HNO formation in cardiomyocytes (Sun et al., 2020c). HNO donor Angeli’s salt significantly ameliorates hygerglycemia-induced myocardial apoptosis, hypertrophy, fibrosis, ROS generation, and cardiac performance in diabetic mice (Sun et al., 2020c). Induction of caveolin-3/eNOS complex may be required for HNO to ameliorate the development of diabetic cardiomyopathy (Sun et al., 2020c). In consistence with this finding, the left ventricular structural remodeling and diastolic dysfunction are limited by chronic administration of HNO donor 1-nitrosocyclohexylacetate (1-NCA) in diabetic FVB/N mice (Cao et al., 2015). It is well known that NO resistance, a state in which the tissue responsiveness to exogenous and endogenous NO is impaired, is frequently occurred in cardiovascular disease states, including diabetic cardiomyopathy (Velagic et al., 2020).

In diabetic rats, acute NO effects induced by diethylamine-NONOate, including vasodilation, myocardial contraction and relaxation, are evidently impaired (Qin et al., 2020). In contrast, isopropylamine-NONOate-induced acute HNO effects are still preserved in the setting of diabetes, and the positive inotropic effects of HNO are obviously elevated in diabetic rat heart (Qin et al., 2020). This intriguing study provides a possible clue that HNO largely circumvents NO resistance in diabetic heart tissues, and supports a possible role for HNO in the management of ischemia and heart failure in diabetes. These above observations suggest that H_2_S and NO enter a redox reaction with each other, resulting in the formation of HNO, a potential way to antagonize the occurrence and development of diabetic cardiomyopathy. However, we can not be too optimistic on the preclinical studies of HNO in diabetic cardiomyopathy due to our insufficient understanding of HNO biosynthesis, chemistry, biology, and pharmacology. Thus, these aspects warrant further interrogation with the emphasis on HNO detection methods, long-lasting donors with safety and efficiency, pharmacological potentials beyond the cardiovascular system.

## Other Mechanisms

Adenosine 5′-monophosphate (AMP)-activated protein kinase (AMPK) is a metabolic master switch that play an indispensable role in energy metabolism and organic lipid homeostasis (Bai et al., 2018). Its activation exerts a beneficial effect in diabetic cardiomyopathy, while its inactivation is involved in cardio-metabolic abnormalities, including diabetes-induced cardiac apoptosis, myocardial fibrosis, and ventricular hypertrophy (Haye et al., 2020). AMPK negatively regulates mammalian target of rapamycin (mTOR), and downregulation of mTOR mediates a host of biological effects induced by AMPK (Liao et al., 2013). A host of studies have highlighted the importance of AMPK inactivation in the pathogenesis of diabetic cardiomyopathy (Haye et al., 2020). On this basis, it is reasonable to hypothesize that H_2_S might protect cardiomyocytes from hyperglycemia stress through AMPK-mediated inhibition of mTOR. Indeed, Wei et al. (2014) have ascertained that GYY4137, a novel H_2_S-releasing molecule, reverses high glucose-induced cell apoptosis, lactate dehydrogenase release, and mitochondrial membrane potential collapse. An AMPK activator, 5-amino-4-imidazole-carboxamide riboside (AICAR), has similar effects to GYY4137 treatment, whereas adenine 9-β-d-arabinofuranoside (an AMPK inhibitor) abolishes GYY4137-mediated cardiac benefits under diabetic conditions (Wei et al., 2014). Most importantly, both GYY4137 and AICAR stimulated AMPK phosphorylation and diminished mTOR phosphorylation in high glucose-challenged H9c2 cells, suggesting that H_2_S ameliorates diabetes-induced cardiac damage in an AMPK/mTOR pathway-dependent manner (Wei et al., 2014).

The presence of cardiac dysfunction in diabetes is accompanied by overload of intracellular calcium in cardiomyocytes (Chen et al., 2020). When cardiomyocytes are excited, increased calcium ion will flow into the cells through L-type Ca^2+^ channels, followed by the release of Ca^2+^ from the sarcoplasmic reticulum (SR) *via* the ryanodine receptor (RyR2), leading to actin–myosin interaction. Cellular calcium overload is closely related with SR dysfunction, such as RyR2-FK506-binding proteins (FKBP12.6) disassociation, downregulation of sarcoplasmic reticulum Ca^2+^ ATPase (SERCA2a) and calsequestrin 2 (CASQ2) (Cheng et al., 2012; Carneiro-Júnior et al., 2014; Roussel et al., 2015; Tomasova et al., 2015). In STZ-induced diabetic rats, the expressions of calcium-handling proteins in the SR, such as FKBP12.6, SERCA2a, and CASQ2 are downregulated, while the concentration of diastolic free calcium was elevated, indicating a calcium leak in diabetic heart tissues (Cheng et al., 2016). However, these abnormalities are attenuated in diabetic rats when treated with NaHS, indicating that H_2_S donors ameliorate diabetic cardiomyopathy-related imbalance in calcium homoeostasis by reversing abnormal expressions of disordered calcium-handling system in the SR (Cheng et al., 2016).

Autophagy plays a critical role in cellular homeostasis by clearing damaged or unnecessary organelles or macronutrients dependent on the lysosomal degradation pathway (Xie et al., 2011). Dysfunction of autophagy is shown to be involved in diabetic cardiomyopathy (Lavandero et al., 2015). It has been revealed that H_2_S is a regulator in autophagy during the process of diabetic cardiomyopathy. In type 2 db/db mouse heart tissues, NaHS facilitates autophagosome content degradation and promotes ubiquitin aggregate clearance *via* autophagy, which might exert its cardiovascular effect in diabetic cardiomyopathy (Wu et al., 2017). It is likely that H_2_S increases autophagic flux to ameliorate diabetic cardiomyopathy.

## H_2_S Protects Against Myocardial Ischemia/Reperfusion Injury in Diabetes

Ischemia/reperfusion cardiac injury is recognized as one of the leading causes of people morbidity and mortality around the world (Yan et al., 2020). Primarily, ischemia/reperfusion leads to the death of cardiomyocytes and increased cardiac infarct size, which can be responsible for up to 50% of the final infarct size (Yellon and Hausenloy, 2007). It has been revealed that H_2_S donors could reduce myocardial ischemia/reperfusion injury by preserving mitochondrial functions and modulation of the signaling pathways of H_2_S by pharmacological agents is likely to prevent ischemia/reperfusion cardiac injury (Andreadou et al., 2020). In addition, the protective roles of H_2_S in myocardial ischaemia/reperfusion injury and cardioprotection by preconditioning or postconditioning have been reviewed (Andreadou et al., 2015). These published findings demonstrated that modulation of endogenous H_2_S production may be of clinical benefit in cardiac ischaemia/reperfusion injury.

Mounting evidence has found that diabetic hearts are more susceptible to ischemic injury under ischemia, hypoxia or anoxia conditions (Hearse et al., 1975; Feuvray et al., 1979; Feuvray and Lopaschuk, 1997). This notion is further supported by clinical studies that diabetic patients complicated with coronary heart disease and post-myocardial infarction might have a worse prognosis with higher incidence of heart failure or sudden death (Jaffe et al., 1984; Gwilt et al., 1985; Stone et al., 1989; Saeid et al., 2018). Studies have shown that hyperglycemia-induced decrease in coronary collateral blood flow and impaired responses of coronary microcirculation to ischemia, might cooperatively lead to exacerbated cardiac dysfunction during myocardial ischemic states (Gu et al., 2003; Badalzadeh et al., 2015). It is likely that hyperglycemia might aggravate myocardial damage in the heart when combined other heart diseases, such as myocardial ischemia-reperfusion injury (Lejay et al., 2016). Interestingly, evidence is emerging that H_2_S is a strong protector against myocardial ischemia-reperfusion injury in diabetes (Jeddi et al., 2020). Therefore, in the sections below, we will provide a comprehensive overview of the therapeutic roles of H_2_S signaling in diabetes-aggravated myocardial infarction or myocardial ischemia-reperfusion injury.

Diabetes not only directly induces cardiac cardiomyopathy (a cardiac disease independent of coronary artery disease, hypertension, and hyperlipidemia), but also worsens cardiac damage in response to myocardial infarction or ischemia/reperfusion (Schramm et al., 2008). To date, various pharmacological interventions are documented to attenuate myocardial ischemia/reperfusion injury in the setting of diabetes (Chin et al., 2017). Among them, H_2_S-based therapies have attracted tremendous attention because of its great potential in limiting cardiac injury during myocardial ischemia-reperfusion under diabetic state (Donnarumma et al., 2017).

Previously, Bridgette et al. had sought to examine whether H_2_S granted a cardioprotection against ischemia-reperfusion injury in the setting of type 2 diabetes (Peake et al., 2013). In this work, they found that H_2_S donor sodium sulfide (Na_2_S) precondition for 24 h or 7 days all improved myocardial injury in db/db diabetic mice (Peake et al., 2013). In an effort to evaluate the signaling mechanism responsible for the observed cardioprotection, they demonstrated that exogenous H_2_S ameliorated myocardial ischemia-reperfusion injury in db/db diabetic mice by activating Nrf2 signaling in an ERK1/2-dependent manner (Peake et al., 2013). Later, the same group further validated that H_2_S provides cardioprotection against myocardial-ischemia reperfusion injury in db/db diabetic mice by activating the ERK1/2 arm of the Reperfusion Injury Salvage Kinase (RISK) pathway (Lambert et al., 2014). Subsequently, several groups have verified that H_2_S-associated protection against ischemia-reperfusion injury in diabetic heart may also involve eNOS-derived NO production (Jeddi et al., 2020), PI3K/GSK3β pathway (Ansari and Kurian, 2020), and preservation of mitochondria (Ansari and Kurian, 2019; Mahalakshmi and Kurian, 2020). These findings provide ample evidence that H_2_S renders cardioprotection against ischemia/reperfusion injury in diabetes. However, more studies are needed to assess whether pre- and post-conditioning the heart with different H_2_S donors could behave differently in terms of ischemia-reperfusion injury limitation.

## Clinical Use of H_2_S Donors in Diabetes

The preclinical studies have provided a robust indication that delivery of H_2_S donors might represent an effective approach for the prevention and treatment of cardiomyopathy and myocardial ischemia/reperfusion injury under diabetes. These studies await further clinical translation. Until May 2021, a total of 15 clinical studies on H_2_S are found at clinicaltrials.gov after typing H_2_S in the search box of “Condition or disease” (https://clinicaltrials.gov/). These studies are performed to test the clinical utilities of H_2_S in various diseases, including chronic kidney disease (ClinicalTrials.gov Identifier: NCT01232257), hypertension (ClinicalTrials.gov Identifier: NCT03179163), heart failure (ClinicalTrials.gov Identifier: NCT01989208), myocardial Infarction (ClinicalTrials.gov Identifier: NCT03829605, NCT02899364), carotid stenosis (ClinicalTrials.gov Identifier: NCT03303534), peripheral arterial disease (ClinicalTrials.gov Identifier: NCT01407172), cardiovascular disease (ClinicalTrials.gov Identifier: NCT02180074). In a clinical trial (ClinicalTrials.gov Identifier: NCT01989208), the authors investigated the effects of H_2_S donor SG1002 on heart failure, and they found that administration of SG1002 increased H_2_S levels and circulating NO bioavailability, but attenuated BNP levels in heart failure patients (Polhemus et al., 2015). However, no results were posted regarding the effects of H_2_S on myocardial infarction, carotid stenosis, peripheral arterial disease, and hypertension. Likewise, the clinical value of H_2_S in diabetic cardiomyopathy and diabetes-aggravated cardiac ischemia/reperfusion injury might attract increased interest in the scientific community. Nevertheless, to the best of our knowledge, no clinical studies have been implemented to test the therapeutic potential of H_2_S in patients with diabetic cardiomyopathy and diabetes-aggravated cardiac ischemia/reperfusion injury. We anticipate that more retrospective and comparative clinical trials will be performed to investigate the therapeutic value of H_2_S in diabetic cardiomyopathy and its progressed heart failure. Of note, more well-designed preclinical or clinical studies are required to assess the safety, effectiveness, pharmacodynamics of H_2_S-based therapeutics on the broader population with diabetic cardiomyopathy and diabetes-aggravated cardiac ischemia/reperfusion injury. Tremendous efforts aiming to translate those basic research results into the clinical arena are underway by using H_2_S-releasing compounds.

## Conclusion and Perspectives

**FIGURE 5:**
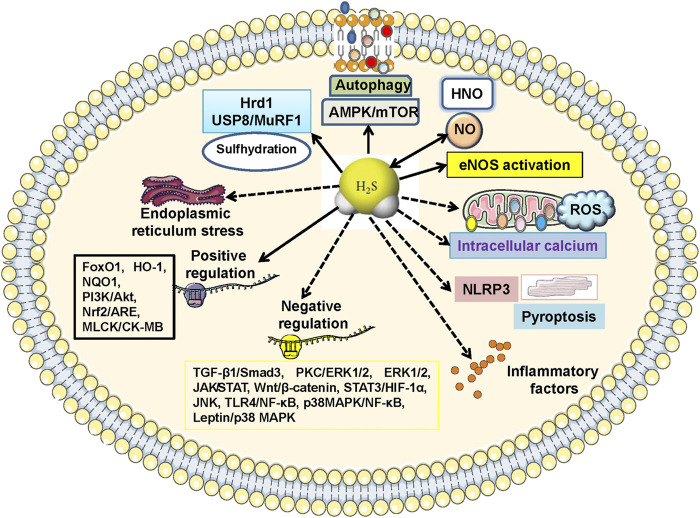
Molecular mechanisms of H2S-mediated cardiac benefits in diabetic cardiomyopathy. The cardioprotective actions of H_2_S on diabetic cardiomyopathy are regulated by multiple signaling cascades. Specifically, 1) inactivation of TGF-β1/Smad3, PKC/ERK1/2, ROS/ERK1/2, JAK/STAT, Wnt pathways, and activation of MLCK/CK-MB, PI3K/AKT, Nrf2/ARE, are responsible for H_2_S-mediabted suppression on myocardial fibrosis and hypertrophy; 2) blockade of endoplasmic reticulum stress, Wnt/*β*-catenin, STAT3/HIF-1*α*, JNK/p38 MAPK, NLRP3, TLR4/NF-κB, p38 MAPK/NF-κB, while stimulation of PI3K/Akt, FoxO1, HO-1, NQO1, contribute to H_2_S-induced effects against cardiomyocyte apoptosis, oxidative stress and inflammation; 3) regulations of intracellular calcium, leptin/p38 MAPK, NLRP3-mediated pyroptosis, AMPK/mTOR, autophagy, eNOS activation, formation of HNO, are also proposed to H_2_S-mediated cardiac benefits in diabetic cardiomyopathy. AMPK, adenosine 5′-monophosphate (AMP)-activated protein kinase; ARE, antioxidant response element; CK-MB, creatine kinase MB isozyme; eNOS, endothelial nitric oxide synthase; ERK1/2, extracellular regulated protein kinase 1/2; FoxO1, forkhead box O1; HIF-1α, hypoxia-inducible factor-1α; HNO, nitroxyl; HO-1, haem oxygenase-1; JAK, Janus kinase; JNK, c-Jun N-terminal kinase; MAPK, mitogen-activated protein kinase; MLCK, myosin light chain kinase; mTOR, mammalian target of rapamycin; NLRP3, nucleotide-binding oligomerization domain-like receptor protein 3; NF-κB, nuclear factor kappa-B; NQO1, NAD(P)H:quinone oxidoreductase 1; Nrf2, nuclear factor erythroid 2-related factor 2; PI3K, phosphatidylinositol 3-kinase; PKC, protein kinase C; ROS, reactive oxygen species; Smad3, SMAD family member 3; STAT, signal transducer and activator of transcription; TGF-β1, transforming growth factor β1; TLR4, toll-like receptor 4.

In this review, we provided the recent advances in our knowledge on the roles of endogenous H_2_S or pharmacologically administered H_2_S donors in diabetes-related cardiomyopathy and myocardial ischemia/reperfusion injury. The present review suggests that H_2_S acts as a cardioprotective mediator in diabetic heart by regulating various signaling pathways (Figure 5). Both endogenously generated H_2_S and exogenously supplied H_2_S donors confer cardioprotection in a variety of settings. Thus, H_2_S might be recommended as a therapeutic agent against diabetic cardiomyopathy and diabetes-aggravated cardiac ischemia/reperfusion injury in pre-clinical studies.

Given that the therapeutic effect of H_2_S on diabetic cardiomyopathy were mediated by multiple signaling pathways, deciphering into the pros and cons of these cellular events induced by H_2_S might facilitate the knowledge of pharmacological activities and drug development of H_2_S. Also, the stable and controllable H_2_S donors should be designed for clinical management of diabetic cardiomyopathy. Eventually, a combination therapy of H_2_S donors with other standard medications might be considered as novel strategies to treat diabetic cardiomyopathy or reduce the risk of heart failure in diabetic subjects.

## Glossary

1-NCA: 1-nitrosocyclohexylacetate
3-MP: 3-mercaptopyruvate
3-MST: 3-mercaptopyruvate sulfurtransferase
AGEs: advanced glycation end products
AICAR: 5-amino-4-imidazole-carboxamide riboside
AMPK: adenosine 5′-monophosphate (AMP)-activated protein kinase
Ang II: angiotensin II
ARE: antioxidant response element
ASC: apoptosis-associated speck-like protein containing A CARD
CASQ2: calsequestrin 2
CAT: cysteine aminotransferase
CBS: cystathionine β-synthase
CHOP: C/EBP-homologous protein
CK-MB: creatine kinase MB isozyme
CO: carbon monoxide
COX-2: cyclooxygenase-2
CSE: cystathionine γ-lyase
DAO: d-amino acid oxidase
eIF2: eukaryotic initiation factor 2
ERK1/2: extracellular regulated protein kinase 1/2
GAPDH: glyceraldehyde-3-phosphate dehydrogenase
GRP78: glucose-regulated protein
H2S: hydrogen sulfide
HFD: high fat diet
HNO: nitroxyl
HO-1: heme oxygenase 1
Hrd1: HMG-CoA reductase degradation protein
IL-1β: interleukin 1β
iNOS: inducible nitric oxide synthase
JAK: Janus kinase
JNK: c-Jun N-terminal kinase
MAPK: mitogen activated protein kinase
MLCK: myosin light chain kinase
MMP-2: matrix metalloprotease 2
mTOR: mammalian target of rapamycin
MuRF1: muscle RING finger-1
NaHS: sodium hydrosulfide
NF-κB: nuclear transcription factor-κB
NLRP3: nucleotide-binding oligomerization domain-like receptor protein 3
NO: nitric oxide
NQO1: Nad(p)h: quinone oxidoreductase 1
Nrf2: nuclear factor erythroid 2-related factor 2
PDE5: phosphodiesterase 5
PERK: pancreatic endoplasmic reticulum kinase
PI3K: phosphatidylinositol 3-kinase
PKC: protein kinase C
RAS: renin–angiotensin system
ROS: reactive oxygen species
RyR2: ryanodine receptor
SERCA2a: sarcoplasmic reticulum Ca2+ ATPase
Smad3: SMAD family member 3
SNP: sodium nitroprusside
STAT3: signal transducer and activator of transcription 3
STZ: streptozotocin
TGF-β1: transforming growth factor β1
TIMP-2: tissue inhibitor of metalloproteinase 2
TLR4: toll-like receptor 4
USP8: ubiquitin specific peptidase 8
VAMP3: vesicle-associated membrane protein 3
